# Growth and neurodevelopment in low birth weight versus normal birth weight infants from birth to 24 months, born in an obstetric emergency hospital in Haiti, a prospective cohort study

**DOI:** 10.1186/s12887-021-02605-3

**Published:** 2021-03-24

**Authors:** Marjorie Hilaire, Xanthi D. Andrianou, Annick Lenglet, Cono Ariti, Kessiane Charles, Sonja Buitenhuis, Daan Van Brusselen, Harriet Roggeveen, Elizabeth Ledger, Rodnie Selva Denat, Lindsay Bryson

**Affiliations:** 1 Médecins Sans Frontières, Port au Prince, Haiti; 2 Médecins Sans Frontières, Plantage Middenlaan 14, 1018DD Amsterdam, The Netherlands; 3 Department of Medical Microbiology, Radboudumc, Nijmegen, Netherlands; 4 Centre for Trials Research, Cardiff University Medical School, Cardiff, UK; 5 Medical Research Council Unit The Gambia at the London School of Hygiene & Tropical Medicine (LSHTM), London, UK

## Abstract

**Background:**

Low birthweight (LBW) infants are at higher risk of mortality and morbidity (growth, chronic disease and neurological problems) during their life. Due to the high incidence of (pre-) eclampsia in Haiti, LBW infants are common. We assessed the anthropometric growth (weight and length) and neurodevelopmental delay in LBW and normal birthweight (NBW) infants born at an obstetric emergency hospital in Port au Prince, Haiti, between 2014 and 2017.

**Methods:**

Infants were followed at discharge and 3, 6, 12, 15, 18, 21 and 24 months of corrected gestational age. At each visit they underwent a physical checkup (weight, length, physical abnormalities, identification of morbidities). At 6, 12, 18 and 24 months they underwent a neurodevelopmental assessment using the Bayley Scale III (motor, cognitive and communication skills). We modelled the trajectories between birth and 24 months of age of NBW compared to LBW infants for weight, length, and raw scores for Bayley III assessments using mixed linear models.

**Results:**

In total 500 LBW and 210 NBW infants were recruited of which 333 (46.7%) were followed up for 24 months (127 NBW; 60.5% and 206 LBW; 41.2%) and 150 died (LBW = 137 and NBW = 13). LBW and NBW babies gained a mean 15.8 g and 11.4 g per kg of weight from discharge per day respectively. The speed of weight gain decreased rapidly after 3 months in both groups. Both groups grow rapidly up to 6 months of age. LBW grew more than the NBW group during this period (22.8 cm vs. 21.1 cm). Both groups had WHZ scores <− 2 up to 15 months. At 24 months NBW babies scored significantly higher on the Bayley scales for gross motor, cognitive and receptive and expressive communication skills. There was no difference between the groups for fine motor skills.

**Conclusion:**

LBW babies that survive neonatal care in urban Haiti and live up to 24 months of age, perform similar to their NBW for weight, length and fine motor skills. LBW babies are delayed in gross motor, cognitive and communication skills development. Further research on the clinical significance of these findings and long term implications of this neurodevelopmental delay is needed.

**Supplementary Information:**

The online version contains supplementary material available at 10.1186/s12887-021-02605-3.

## Background

Low birthweight (LBW) in neonates is defined as a birth weight of less than 2500 g by the World Health Organization [1]. LBW classification is determined at birth and based on the absolute weight of the baby at birth regardless of gestational age. It is multifactorial in nature and can be caused by preterm delivery or restricted foetal (intra-uterine) growth [2, 3]. The latter can also result in the babies being small for gestational age (SGA), which is most commonly defined as a baby with a weight below the 10th percentile for the gestational age [4]. Numerous studies have identified the factors, in addition to prematurity, that contribute to LBW including deprived socio-economic conditions of the mother (poor nutrition, poor access to care, high prevalence of infections and high prevalence of pregnancy complications), and maternal health during pregnancy (nutrition, diet, use of alcohol/drugs/tobacco, infections and presence of hypertension and diabetes) [2].

The survival rates of LBW infants have improved with improved clinical management. However, this group of infants remains at risk of higher mortality and morbidity during the neonatal period (i.e. within 28 days after birth) and thereafter. LBW is known to be associated with subsequent health issues such as poor anthropometric growth in childhood and higher incidence of non-communicable disease in adulthood such as hypertension, stroke, diabetes, and hypercholesterolemia and with long term neurological problems (physical and learning disabilities) [5–7]. Several studies carried out in high resource settings have identified that surviving preterm and/or LBW infants (compared to NBW infants) followed up after birth (up to a maximum of 11 years) suffered from cerebral palsy, visual disability (blindness), deafness, problems with walking and poor performance on neurodevelopmental assessments [6, 8–13].

Haiti is the poorest country in the Americas with recent estimates that 59% of Haitians live under the national poverty line [14]. The prevalence of pre-eclampsia in pregnant women (an important cause of prematurity and delivery of small-for-gestational age infants) in Haiti is high, estimated at 18% in 2005 [15, 16]. The ‘Centre de Référence des Urgences Obstetricales’ (CRUO) was established by Médecins Sans Frontières (MSF) in Port au Prince (the capital) in 2010 to manage the high number of complicated deliveries (especially women suffering from pre-eclampsia). A neonatal ward was also established to care for the high burden of LBW and/or premature neonates born to women with complicated pregnancies. Between January 2013 and June 2018, out of the 31,509 maternal admissions in CRUO, 34.9% were women that had (pre-) eclampsia and of the 24,983 deliveries documented in the hospital, 11,008 (44.1%) were LBW babies (MSF unpublished data).

Public health structures (or organisations) able to manage long term care for infants with morbidities resulting from their prematurity or LBW are extremely limited in Haiti. Assessing neurodevelopmental outcomes in these infants may help clinical decisions to be made thoughtfully for neonatal care and ongoing support in humanitarian settings. We undertook a prospective cohort study to describe and compare the anthropometric growth (in terms of weight and length) and neurodevelopment between LBW and normal birthweight (NBW) infants born at CRUO. We hypothesized that growth and neurodevelopment outcomes would be poorer in the LBW group compared to the NBW group within 24 months after their birth.

## Methods

### Neonatal care provided at CRUO

CRUO was an obstetric specialty hospital providing care for complicated pregnancies, deliveries and neonates when it was needed. It was opened in 2013 and closed in July 2018.

A pediatric healthcare team (midwives, nurses, nurse-assistants and doctors) provided neonatal care at CRUO. Midwives and nurses were trained in basic neonatal life support (NLS). Neonatal care included treatment for the following morbidities: hypothermia (incubators, heating lamps), hypoglycemia (IV or enteral glucose), respiratory problems (low flow oxygen through continuous positive airway pressure;CPAP), infections (antibiotics), apneas of prematurity (caffeine), jaundice (phototherapy) and gastrointestinal problems (early enteral feeding – either by nasogastric tubes or orally). For well or improved LBW/premature neonates a well-functioning Kangaroo Mother Care (KMC) ward was available. Establishing breastfeeding was supported in all gestations and expressed breast milk given through a variety of means (including a nasogastric tube; NGT). Routine neonatal care was available to all neonates born at CRUO, including the administration of vitamin K for those with bleeding disorders and the administration of hepatitis B, polio and Bacillus Calmette–Guérin (BCG) vaccinations to all healthy/recovered neonates prior to discharge from the hospital.

### Study design

We conducted a prospective cohort study of infants born in CRUO hospital with participants followed up from birth and post discharge at 3, 6, 12, 15, 18, 21 and 24 months corrected for gestational age.

### Participants

All neonates born in the hospital between October 2014 and May 2015 were eligible for inclusion in the study. They were excluded if presenting with any major and obvious congenital malformations at birth (i.e. anal atresia, spina bifida etc.). They were also excluded if they were abandoned by their parents or caretakers at the hospital or if their parents or caretakers did not consent to inclusion in the study.

Infant recruitment was done by the study team with the parents and/or caretakers of the neonates. The study team was alerted each time a new baby was born and then explored the options for inclusion into the study. In order to maintain a feasible number of babies to be seen during follow up visits, the team included no more than 10 infants per day.

### Data measurements and collection

Evaluation of LBW was done through measurements at birth, but also upon admission to the neonatal unit. The weight at birth was measured using a digital Seca scale. Gestational age of the neonate was measured initially in the emergency department or delivery room either by fundal height, ultrasound or by history given by mother. After birth, the gestational age was additionally confirmed using the Ballard score for those babies who were admitted to the NICU or pediatric ward. The correction for gestational age was done by subtracting the measured gestational age at birth from 40 weeks (as the standard gestational age for a term birth). The difference was then added to the babies’ true date of birth to estimate what the date of birth would have been at term. This corrected birthdate was used to schedule follow up visits. We defined low Apgar at birth as any neonate that scored below seven at one, five and 10 min.

All infants recruited into the study had their medical charts and their mother’s medical charts reviewed prior to discharge. At discharge, 3, 6, 12, 15, 18, 21 and 24 months of corrected gestational age, the infants underwent a physical checkup (weight, length, physical abnormalities, and identification of morbidities). For the visits of 6, 12, 18 and 24 months they also underwent a neurodevelopmental assessment using the Bayley Scale III [17].

During follow up visits the following medical conditions revealed by physical examination were treated by the study team: simple infections not requiring hospital admission, uncomplicated malaria, clinically suspected gastrointestinal helminthic infections or feeding difficulties (inability to latch/suck, regurgitation). Any suspected medical condition that required inpatient paediatric, orthopaedic or surgical services, were referred to specialised care when possible (the costs for referral appointments were not covered by the study team). If between follow up visits study participants were sick, they could also consult the study team for free medical care or referral when possible.

We conducted weight assessments using a Salter scale until the infants were able to stand by themselves. Thereafter we used Seca 725 electronic weight scales. Length measurements at birth, at discharge and at six months were done using flexible, non-stretch tape measures. All further length measurements were conducted using a wooden infant length board. Infants were placed on their back and their length was measured from the top of their head to the sole of their feet. Length measurements with a measuring tape are not standard practice for six-month-old infants, therefore we calibrated these measurements to the equivalent wooden infant board measurement using a corrective factor derived from a new set of 40 infants (20 LBW and 20 NBW) for whom we took measurements at discharge, three and six months using both the measuring tape and measuring board approach. A linear regression model was used to estimate the corrective factor.

Neurodevelopment was measured at four time points (6, 12, 18 and 24 months corrected gestational age) using the Bayley Scales of Infant and Toddler Development (Third Edition, Pearson, San Antonio, USA). The Bayley III is used to determine developmental delay but has never been validated for the Haitian population. The test comprises of three index scores: 1) motor skills 2) cognitive skills and 3) language. In children under two years of age, the motor scale score consists of assessment of fine motor skills (eye movement coordination, reaching and grasping for objects, grasping of objects with whole hand and gradually between thumb and forefinger) and gross motor skills (sitting, crawling and walking). The cognitive score assesses play skills (solitary non-relational play and social fantasy play) and information processing (attention to novelty, habituation, memory and problem solving). The language score assesses both receptive (responding to sounds and voices, discriminating between sounds, localizing sounds and ability to comprehend and respond appropriately to words and requests) and expressive communication skills (the infant’s ability to vocalise, ability to do one-word approximations, naming of objects/pictures/actions, ability to communicate wants and needs, ability to respond to questions, ability to use multiple word sentences and the ability to combine words and gestures).

Data was collected by a team of pediatricians (three) and pediatric nurses (three) over a period of three years. All chart review and medical visits were documented in structured questionnaires for those visits. Data were transferred from paper format to electronic databases (password protected) which had been created in Epi Data 3.1 (Odense, Denmark) by a specifically trained study nurse. We also used the software Psychmotor Corp for the entry of data around the Bayley III assessments.

All pediatricians and pediatric nurses were trained on the administration of the Bayley scales by a certified Bayley scale trainer. This training took place for one week prior to the start of the study and a refresher training (plus more advanced modules) were covered one year after the start of the study. In order to minimize differences in scoring between the teams of pediatricians and pediatric nurses, repeated quality control sessions were done at the start of each follow up round. One team would film the child under evaluation on all Bayley scale aspects. The other two teams would then repeat the Bayley scale assessments (blinded from the other teams) by reviewing the film footage. Discrepancies were discussed with the Study Coordinator and a pediatrician from CRUO (not part of the study team) to agree on best scoring practices. This was repeated for up to five infants at the start of each follow up visit round.

### Sample size

As there is very limited published evidence using a similar cohort of infants in a low resource setting, establishing reliable assumptions for the calculation of a sample size to estimate growth and neurodevelopment trajectories was challenging. Similar studies carried out in high resource settings generally include thousands of babies from birth cohorts, which was not a feasible option in our setting. Without existing evidence from Haiti, we therefore based our assumptions for mortality in LBW (and sub-categories of LBW) and NBW infants on data collected at CRUO during 2013 and on studies on mortality in infants born in Nigeria, Kenya and Tanzania [18–20]. We used OpenEpi for all sample size calculations (https://www.openepi.com/Menu/OE_Menu.htm).

In order to be able to have a sufficient sample size to identify additional risk factors within the LBW infants discharged from CRUO we assumed that the ‘exposure’ occurred in different categories of LBW (very LBW and extremely LBW, where VLBW = < 1000 g and ELBW = < 1500 g) and the outcome was mortality. During 2013 in CRUO, 1047 infants were born that were < 2.5 kg (i.e. 47% out of all infants born) and the remainder were normal weight (therefore a ratio of 1:1 for LBW and NBW). Out of this group of infants, 445 (42.5%) weighed between 1.75–2.5kgs, and 602 (57.5%) weighed < 1.75kgs (i.e. 1045 VBLW and ELBW combined). If we consider the first group as LBW and the second group as VLBW and ELBW, then the ratio of non-exposed to exposed would be 0.74 (around 3 non-exposed to 4 exposed). We assumed: 1) two-sided significance level of 95%; 2) power of the study is 80%; 3) ratio of unexposed/exposed is 0.75; 4) expected relative risk is 3; 5) expected proportion of outcome in unexposed group is 5%. For the outcome of mortality in sub-categories of LBW we calculated 191 infants in the exposed group (VLBW and ELBW infants) and 143 infants in the unexposed group (LBW excluding VLBW and ELBW infants) for a total of 334 infants. Assuming a default rate in the LBW group of approximately 35% (assuming 334 infants remain in the study) and 25% in the NBW group (assuming 160 remain in the study) we aimed to enroll 500 LBW infants and 210 NBW infants.

### Data analysis

We summarised the characteristics of the study population using number and percentage for categorical variables and mean and standard deviation for continuous variables. We compared differences of all sociodemographic variables for the infants between the NBW and LBW groups using Chi-square tests for categorical variables and two independent t-test for continuous variables. We calculated weight gain (in grams) per day per kilogram (kg) of last weight measurement for each infant enrolled in the study. Z-scores for weight-for-height (WHZ) were calculated using the WHO standards and described for the NBW and LBW for each visit to determine the level of malnutrition in the study population.

The primary outcomes were the growth trajectories between birth and 24 months of age of NBW compared to LBW infants in terms of weight (grams), length (centimeters), and raw scores for Bayley III assessments for motor, cognitive and communication skills. For the weight, length and raw scores of the Bayley III assessments’ trajectories, we fitted a linear mixed model to estimate the length, weight and Bayley III assessment raw scores of each individual infant in the NBW and LBW group. Thus, the weight, length and Bayley III assessment raw scores at each follow up visit of the individual were the dependent variables.

We modelled the trajectories by considering time since birth as a series of linear spline components [21]. We chose to place the linear splines at specific time points (“knots”). For the length and weight trajectory, we placed the knots at 3 months and 12 months to facilitate the early, middle and late growth periods. The Bayley III assessments’ scores had measures at fewer time points, and this meant that the model required fewer knots. For the motor skills scales a single knot was placed at 12 months and for the cognitive skills scale a single knot was placed at 18 months. By using linear splines, the interpretation of the parameters was simply the slope of the line between the knots. This parametrization of the model allowed us to examine changes in slope between different time-periods and thus assessed if rate of change itself was changing across time. For example, in the length and weight models placing knots at 3 and 12 months of age allows us to estimate the rate of growth in three time-periods, namely: 0–3 months, 3–12 months and 12–24 months as well as measure if that rate of growth is different in those time periods.

We included interaction terms between the LBW and NBW groups and the spline terms in order to reflect the shape of the curves for weight, length, cognitive and language skills, fine and gross motor skills for each group (NBW or LBW). We assumed a random effects covariance structure that allowed each child to have their own latent intercept and linear spline growth trend. The models were also adjusted for sex (supplementary information). We did not adjust the models based on the prematurity of individual babies as we know gestational age has limited precision in this setting (where it was estimated using numerous methods).

The estimated values of a child’s length, weight and raw Bayley III scores and 95% confidence intervals (95%CI) were calculated from the linear mixed regression models at each six months of age from birth to 24 months of age (depending at which age they would be recorded). Estimated values were made by birthweight group (NBW vs LBW), and sex of the infant when relevant. In some cases, the could not be calculated where data were too sparse for that particular time point. Estimated values and 95%CI of the outcome variables at specific time points (0, 6, 12, 18 and 24 months) were calculated from the results of the relevant linear mixed models using linear combinations (contrasts) of the parameter estimates and associated variance-covariance-matrix. Absolute differences between outcomes at specific time points and *p* values were also calculated from the linear mixed model results in the same way.

Data cleaning was conducted using STATA13 and data analysis using R (version 3.6.2) [22]. The ‘emmeans’ and ‘ggpredict’ packages in R were used to calculate the estimated values and 95%CIs, absolute differences, standard errors and *p*-values [23, 24].

## Results

### Description of study participants

**Fig. 1:**
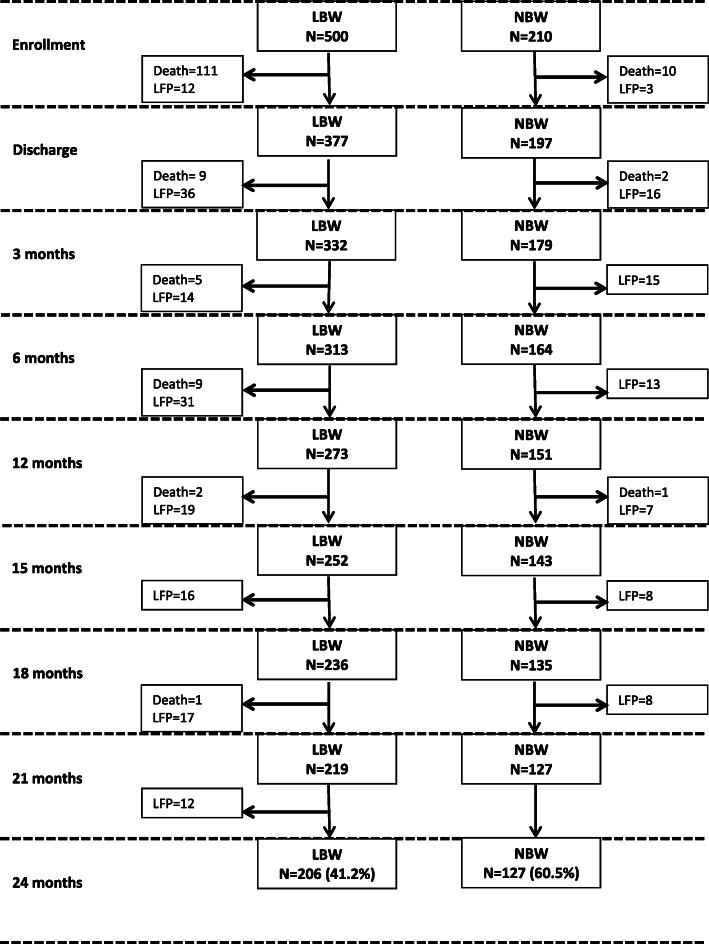
Study participant flow in the study [LBW = low birthweight; NBW = normal birthweight, LFP = lost to follow up]; total deaths = 150 (LBW = 137; NBW = 13); total LFP = 227 (LBW = 157; NBW = 70)

We enrolled 710 infants in the study between October 2014 and May 2015; 500 were LBW and 210 were normal weight. Of the 500 LBW infants that were enrolled in the study, 206 (41%) were followed up to the 24-month period. Between enrollment and the last follow-up visit, 137 (27%) died and 157 (31%) were lost to follow up (LTFU). Of the 210 NBW infants enrolled in the study, 13 died (6%) and 70 were lost to follow-up (33%) and 127 (60%) were followed up to the 24-months’ time-period (Fig. 1).

Out of the total deaths (*n* = 170), the majority (121, 71%) died before the neonates were discharged from the hospital during a time in which the pediatric unit was battling a hospital-acquired outbreak of sepsis caused by a multi-drug-resistant *Klebsiella pneumoniae* [25] (Fig. 1). For 120 neonates for whom the cause of death was available, 62 (51.6%) died from sepsis/septic shock, 13 (10.8%) died from severe prematurity, nine (7.5%) died from hyaline membrane disease and six (5%) from perinatal asphyxia/hypoxia.

Of the infants enrolled, more LBW infants were female compared to NBW infants (58.4% vs. 42.4%) (Table 1). The mean gestational age in LBW babies was approximately five weeks shorter than in NBW babies (LBW: 35 weeks [IQR: 32–39], NBW: 40 weeks [39–40]; *p* < 0.001). According to the prematurity categories, 16 NBW babies (8.2%) were born with late or moderate prematurity (32 to 36 weeks gestational age) (Table 1). LBW babies were born in all gestational age categories with 166 (33.2%) born full term (Table 1). Twenty-two of the LBW babies (16.5%) were born weighing < 1000 g (Table S1). There were 87 infants in the LBW group (17.4%) that were classified as twins compared to 16 babies (7.6%) in the NBW group (*p* = 0.001; Table 1). Finally, LBW infants were more likely to have been hospitalized after birth and to have died (78.0% vs. 52.4%; *p* < 0.0001, Table 1).

**Table 1:** Estimated weight from linear mixed models and 95% confidence intervals (in grams) at birth, 6, 12, 18 and 24 months for NBW and LBW infants adjusted for age

Age (months)	NBW			LBW	Absolute Difference (SE)	***p***-value
	Estimated weight (g)	95% CI	Estimated weight (g)	95% CI		
**0**	2777.4	2696.0–2858.8	1578.2	1516.7–1639.5	1199.3 (52.0)	< 0.001
**6**	7044.7	6905.2–7184.2	5963.2	5861.5–6064.9	1081.5 (88.1)	< 0.001
**12**	8872.2	8681.7–9062.7	7998.8	7859.4–8138.2	873.4 (120.4)	< 0.001
**18**	10,116.2	9917.5–10,314.9	9252.8	9107.9–9397.7	863.4 (125.5)	< 0.001
**24**	11,360.3	11,133.2–11,587.3	10,506.8	10,340.4–10,673.2	853.5 (143.6)	< 0.001

*NBW* normal birth weight, *LBW* low birth weight, *SE* standard error

### Morbidities

We identified 22 infants with suspected cardiopathy (LBW = 16 and NBW = 6), 10 with macrocephaly (LBW = 9 and NBW = 1), six with microcephaly (LBW = 3 and NBW = 3), seven with a suspected chromosomal abnormality (LBW = 5 and NBW = 2) and three infants with club foot (LBW = 2 and NBW = 1). There were no significant difference between the LBW and NBW for any of these. Three infants with suspected cardiopathy (all LBW) and two with a chromosomal abnormality (both LBW) died during the study.

The most commonly less severe morbidities were skin-related (439/3806 visits, 11.53%) and flu-like illness syndrome (451/3806 visits, 11.85%). Other commonly identified morbidities included acute respiratory infections (including bronchitis, bronchiolitis and suspected pneumonia)(211/3806, 5.54%), anemia (115/3806 visit, 3.02%), gastrointestinal symptoms (116/3806, 3.05%), suspected urogenital infection (84/3806, 2.21%) and conjunctivitis (62/3806, 1.63%). There were no significant differences in morbidity rates between LBW and NBW groups (*p* > 0.05 for all categories).

### Weight

The mean weight at birth for the LBW group was 1981 g (SD:460.1) and for the NBW group 3009 g (SD:407.2). At 24 months the mean weight of the LBW and NBW groups was 10,912 g (SD:1438.0) and 11,464 g (SD:1388.8), respectively (Fig. S1). In the first three months of life, LBW babies increased their weight with 15.8 g (SD:4.7) per day per kilogram (kg) of weight from discharge. Comparatively, NBW gained a mean of 11.4 g per kg of weight from discharge per day (Table S1) in the first three months of life. The speed of weight gain per day per kg decreased rapidly after the third month of life in both groups and averaged to about 1 g per day per kg from the previous visit from 12 months of follow up until the end of the study period (Fig. S2).

**Fig. 2:**
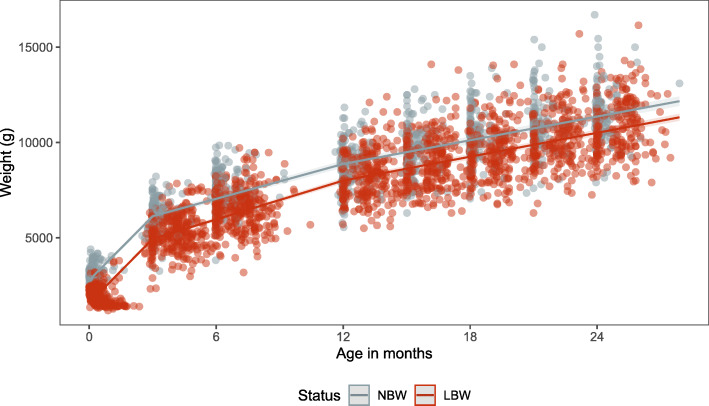
Weight (cm; dots) and estimated weight (lines) for the NBW and LBW groups

The rapid growth is reflected in the regression models that calculated the estimated weights each six months for 24 months of age for the NBW and LBW group (Table 1 and Fig. 2). Estimated weight gain was faster in the first six months for both NBW and LBW infants as demonstrated by the steep slope of the estimated curve until the knot at six months (Fig. 2). In the model NBW babies are significantly heavier than LBW babies at birth (1200 g heavier; Table 1). They remain significantly heavier at each age up to 24 months of follow up but the difference in weight reduces to below 900 g at 24 months of age (Table 1). Male infants are estimated to be heavier compared to females in both the NBW and LBW study groups and in all age groups (Table S2).

### Length

**Fig. 3:**
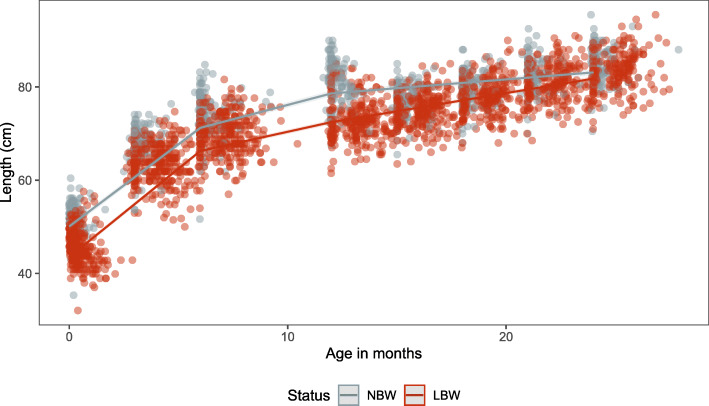
Length (cm; dots) and estimated length (lines) for the NBW and LBW groups

At birth, LBW babies were shorter compared to NBW babies (mean length: 45.9 cm vs. 51.3 cm). The difference in mean length between the LBW and NBW decreased by the 24 month follow up period (83.4 cm vs. 85 cm) (Fig. S2). In the regression models, LBW are estimated to be 6.6 cm shorter compared to NBW babies at birth (43.5 cm; 95%CI: 43.1–44.0 versus 50.1 cm; 95%CI: 49.5–50.7) (Table 2). Following this, both the NBW and LBW undergo an estimated rapid growth spurt up to the knot at 6 months of age (Fig. 3) with LBW estimated to grow more than the NBW group during this period (22.8 cm vs. 21.1 cm). For the 24 month follow up period, NBW are estimated to remain significantly taller than LBW babies (Table 2). The length difference however does reduce to 1.3 cm by the 24 months estimated length measurement (Table 2). Male babies in the NBW and LBW groups are estimated to be taller than females in their birthweight group for the duration of the 24-month study period (Table S3).

**Table 2:** Estimated length from linear mixed models and 95% confidence intervals (in grams) at birth, 6, 12, 18 and 24 months for NBW and LBW infants

Age (months)	NBW		LBW		Absolute Difference (SE)	***p***-value
	Estimated length (cm)	95% CI	Estimated length (cm)	95% CI		
**0**	50.1	49.5–50.7	43.5	43.1–44.0	6.6 (0.4)	< 0.001
**6**	71.2	70.7–71.8	66.3	65.9–66.7	5.0 (0.3)	< 0.001
**12**	78.6	78.0–79.2	72.5	72.0–72.9	6.2 (0.4)	< 0.001
**18**	80.9	80.3–81.4	77.1	76.7–77.5	3.7 (0.4)	< 0.001
**24**	83.1	82.5–83.8	81.8	81.3–82.3	1.3 (0.4)	0.001

*NBW* normal birth weight, *LBW* low birth weight, *SE* standard error

### Weight for height

**Fig. 4:**
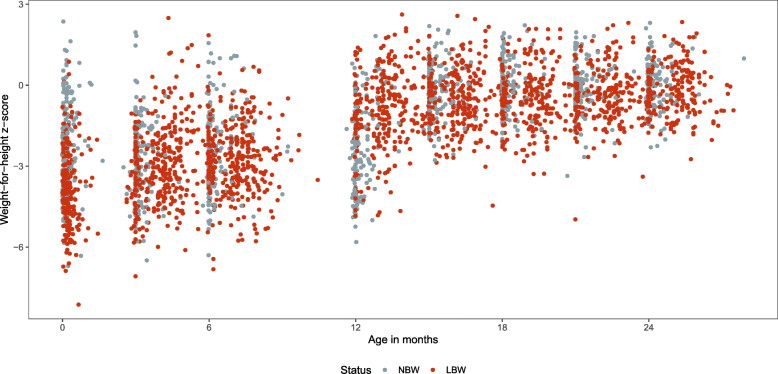
Weight-for-Height z scores for the NBW and LBW groups

We explored Weight for Height Z-scores for both groups of babies in the cohort. In both groups, the WHZ scores only reached above − 2 after 15 months of age (Fig. 4).

### Bayley scales raw scores

Table 3 shows the mean and median raw scores obtained by the NBW and LBW at each of the follow up visits. From the models, the estimated score for raw scores for gross motor skills increases from 25.7 (95%CI: 24.7–26.7) to 54.7 (95%CI: 53.7–55.7) between 6 and 24 months of age for NBW babies and from 21.2 (95%CI: 20.4–22.0) to 52.3 (95%CI: 51.6–53.1) for the LBW babies (Table 4). The estimated raw scores for gross motor skills are significantly higher across each follow up visit in NBW babies compared to LBW babies (Table 4) even though absolute differences are minimal at the end of the study period.

**Table 3:** Bayley Scales III mean and median raw scores for gross motor skills, fine motor skills, cognitive skills, receptive communication skills and expressive communication skills at follow-up visits corresponding to age corrected for gestational age

Bayley scale	Follow up visits corresponding to age (months)	NBW			LBW		
		n	Mean (SD)	Median [IQR]	n	Mean (SD)	Median (IQR)
**Gross Motor skills**	**6**	179	26.5 (5.3)	27 [1, 38]	332	24.2 (5.6)	25 [0, 38]
	**12**	164	40 (6.6)	40 [4, 51]	312	38.6 (6.3)	39 [2, 52]
	**18**	143	50.2 (5.9)	52 [8, 57]	251	48 (7.3)	50 [3, 56]
	**24**	127	54.1 (6.3)	55 [5, 62]	218	53.4 (4.7)	55 [17, 61]
**Fine motor skills**	**6**	179	19.9 (3.7)	21 [5, 27]	312	12.9 (3.6)	14 [3, 19]
	**12**	164	28.8 (4)	30 [6, 34]	251	17.4 (5)	17 [5, 28]
	**18**	143	33.5 (4)	34 [7, 39]	218	24.2 (6.9)	25 [5, 39]
	**24**	127	36.9 (4.5)	37 [12, 44]	312	12.9 (3.6)	14 [3, 19]
**Cognitive skills**	**12**	164	39.6 (7.7)	41 [5, 56]	312	40.1 (7.3)	41 [3, 52]
	**18**	143	50.9 (8.2)	53 [10, 63]	251	48.9 (7.8)	51 [10, 61]
	**24**	127	56.9 (8.6)	59 [14, 69]	218	55.3 (7.6)	56.5 [23, 67]
**Receptive communication skills**	**12**	164	13.1 (2.2)	13 [6, 18]	312	12.8 (2.3)	13 [3, 17]
	**18**	143	18.5 (3.7)	19 [9, 26]	251	18.5 (3.7)	19 [6, 26]
	**24**	127	24.2 (4.4)	25 [6, 31]	218	23.4 (4.5)	25 [11, 39]
**Expressive communication skills**	**12**	164	12.7 (3.4)	13 [2, 19]	312	12.9 (3.6)	14 [3, 19]
	**18**	143	18.8 (4.9)	19 [4, 29]	251	17.4 (5)	17 [5, 28]
	**24**	127	25.5 (7)	26 [5, 39]	218	24.2 (6.9)	25 [5, 39]

*NBW* normal birth weight, *LBW* low birth weight

**Table 4:** Estimated Bayley Scales III raw scores from linear mixed models and 95% confidence intervals for gross motor skills, fine motor skills, cognitive skills, receptive communication skills and expressive communication skills

Bayley scale	Age (months)	NBW		LBW		Absolute Difference (SE)	p-value
		Estimated score	95%CI	Estimated score	95%CI		
**Gross Motor skills**	**6**	25.7	24.7–26.7	21.2	20.4–22.0	4.5(0.7)	< 0.001
	**12**	40.6	39.6–41.6	37.3	36.6–38.1	3.3(0.6)	< 0.001
	**18**	47.7	46.8–48.6	44.8	44.2–45.5	2.8(0.6)	< 0.001
	**24**	54.7	53.7–55.7	52.3	51.6–53.1	2.4(0.6)	< 0.001
**Fine motor skills**	**6**	19.4	18.8–20.0	17.4	16.9–17.9	1.9 (0.4)	< 0.001
	**12**	28.9	28.3–29.5	28.8	28.3–29.2	0.1 (0.4)	0.70
	**18**	32.9	32.4–33.4	32.3	31.9–32.7	0.6 (0.3)	0.06
	**24**	36.8	36.2–37.4	35.8	35.4–36.3	1.0 (0.4)	0.008
**Cognitive skills**	**12**	39.2	37.9–40.5	38.1	37.1–39.2	1.0 (0.9)	0.22
	**18**	50.4	49.1–51.8	47.8	46.7–48.9	2.6 (0.9)	0.003
	**24**	56.3	54.9–57.6	53.9	53.0–54.9	2.3 (0.8)	0.005
**Receptive communication skills**	**12**	12.9	12.3–13.5	11.7	11.2–12.2	1.2 (0.4)	0.003
	**18**	18.2	17.6–18.8	17.5	17.0–18.0	0.7 (0.4)	0.07
	**24**	23.8	23.2–24.4	22.4	22.0–22.8	1.4(0.4)	< 0.001
**Expressive communication skills**	**12**	12.5	11.6–13.3	12.0	11.3–12.7	0.5 (0.6)	0.43
	**18**	18.4	17.5–19.3	16.3	15.6–17.1	2.1 (0.6)	< 0.001
	**24**	25.1	24.2–26.0	22.7	22.1–23.4	2.3 (0.5)	< 0.001

*NBW* normal birth weight, *LBW* low birth weight

**Fig. 5:**
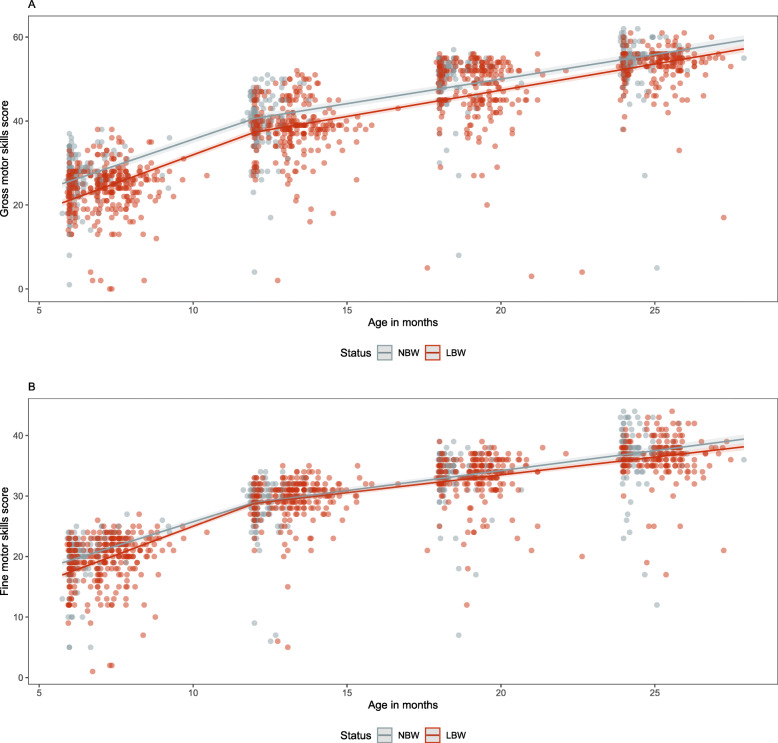
Gross and fine motor skills scores (dots) and estimated values (lines) for the NBW and LBW groups (**a**, **b**)

The estimated score for raw scores for fine motor skills increases from 19.4 (95%CI: 18.8–20.0) to 36.8 (95%CI: 36.2–37.4) between 6 and 24 months of age for NBW babies and from 17.4 (95%CI: 16.9–17.9) to 35.8 (95%CI: 35.4–36.3) for the LBW babies (Table 4). The difference between the NBW and LBW estimated raw scores for fine motor skills are only significant at 6-months of age. After this period, the confidence intervals for estimated raw fine motor skills scores overlap (Fig. 5). There are no differences in estimated raw scores for gross or fine motor skills in females compared to males in NBW and LBW babies (Table S4 and S5).

**Fig. 6:**
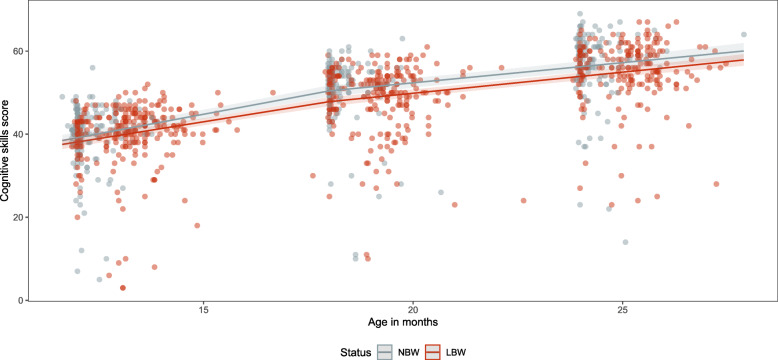
Cognitive skills scores (dots) and estimated values (lines) for the NBW and LBW groups

The estimated raw scores for cognitive skills from 12 to 24 months of age increase from 39.2 (95%CI: 37.9–40.5) to 56.3 (95%CI: 54.9–57.6) in NBW babies and from 38.1 (95%CI: 37.1–39.2) to 53.9 (95%CI: 53.0–54.9) in LBW babies (Tables 4 and 5). There are no significant differences between either of the two study groups (Fig. 6) even if the absolute estimated raw scores for cognitive skills are higher in the NBW babies. NBW and LBW male babies consistently had higher estimated raw scores for cognitive skills compared to their female NBW and LBW counterparts (Table S4 and S5).

**Table 5:** Enrollment characteristics of LBW and NBW study participants

		NBW		LBW		***p***-value
		*N* = 210		*N* = 500		
		*N*	*%*	*N*	*%*	
**Gestational age [mean (SD)]**		39.3 (1.5)		35.1 (3.7)		< 0.001
**Gestational age categories**	Extremely preterm (≤28 weeks)	0		23	4.6	< 0.001
	Preterm (29–31 weeks)	0		59	11.8	
	Moderate prematurity (32–33 weeks)	2	1.0	85	17.0	
	Late preterm (34–36 weeks)	14	6.7	167	33.4	
	Full term (≥37 weeks)	194	92.4	166	33.2	
**Gender**	Female	89	42.4	292	58.4	< 0.001
	Male	121	57.6	208	41.6	
**Birthweights [mean (SD)]**		3081.4 (407.2)		1867.1 (460.1)		< 0.001
**Birthweight [median IQR]**		2970 (1720–4400)		1990 (1185–3800)		
**Birthweight categories**	< 1000 g	0		24	4.9	< 0.001
	1000–1499 g	0		79	16.1	
	1500–1999 g	0		166	33.9	
	2000–2999 g	0		221	45.1	
	≥2500 g	205	100	0		
**Low Apgar at 5 or 10 min**		38	18.5	146	29.7	0.002
**Twin**		16	7.6	87	17.4	0.001
**Hospitalisation after birth**		110	52.4	390	78.0	< 0.001
**Dropout within 24 months**	Death	13	15.7	137	46.6	< 0.001
	Lost to follow up	70	84.3	157	53.4	

*NBW* normal birth weight, *LBW* low birth weight

**Fig. 7:**
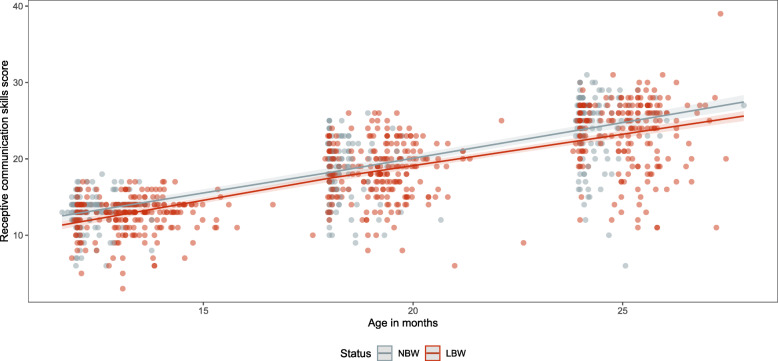
Receptive communication skills scores (dots) and estimated values (lines) for the NBW and LBW groups

**Fig. 8:**
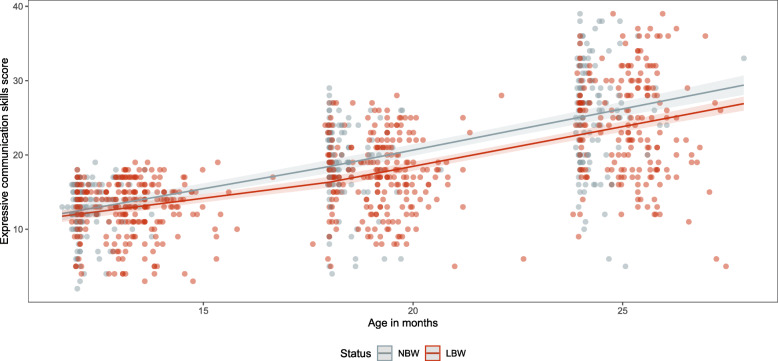
Expressive communication skills scores (dots) and estimated values (lines) for the NBW and LBW groups

Between 12 and 24 months of age, the estimated raw scores for receptive communication skills increased from 12.9 (95%CI: 12.3–13.5) to 23.8 (95%CI: 23.2–24.4) in NBW babies and from 11.7 (95%CI: 11.2–12.2) to 22.4 (95%CI: 22.0–22.8) in LBW babies (Table 4). The estimated raw scores for receptive communication skills are observed to be higher (confidence intervals do not overlap) in the NBW group compared to the LBW group at both 12 and 24 months of age (Fig. 7). Between 12 and 24 months of age, the estimated raw scores for expressive communication skills increased from 12.5 (95%CI: 11.6–13.3) to 25.1 (95%CI: 24.2–26.0) in NBW babies and from 12.0 (95%CI: 11.3–12.7) to 22.7 (95%CI: 22.1–23.4) in LBW babies (Table 4). The difference in estimated raw scores for expressive communication is not significant between NBW and LBW babies at 12 months of age but becomes significantly different at 18 and 24 months of age (Fig. 8). The estimated raw receptive communication skills are very similar between the female and male babies in both the NBW and LBW group (Table S4 and S5). The estimated raw scores for expressive communication skills are higher in male babies in the NBW and LBW compared to their female counterparts (Table S4 and S5).

## Discussion

We have described the first ever cohort of Haitian NBW and LBW babies followed for up to 24 months of age in an urban low resource setting. We have shown that LBW babies in this context had a higher risk of death in the neonatal period compared to NBW babies due to their exposure to an ongoing outbreak of *K. pneumonia* as well as having other morbidities associated with prematurity. LBW babies gained weight more rapidly compared to their NBW counterparts in the first three months of life, which can be seen as catch-up growth. Such catch up growth has been shown to be associated with improved renal and general health [26, 27]. Even so, we estimated that at 24 months of life, LBW babies had lower weight measurements compared to NBW babies. The rapid growth in length in the first three months of life was seen in both NBW and LBW babies, with LBW estimated to be almost caught up to the NBW group at 24 months of age.

The rapid weight gain in the first three months of life in the LBW baby group (15.8 g/kg/day) was close to that observed in extremely LBW babies included in a recent study in South Africa [28]. It is also remarkably close to the standard growth per kilogram per day of 15 g/kg/day [29]. The comparatively slower growth per day (11.4 g/kg/day) in the NBW group (most of whom were never admitted to the hospital post birth), could be partially explained by the fact that LBW babies were followed more closely (during hospitalisation) in terms of breastfeeding and nutrition practices of their mothers as a part of the KMC component in the hospital. Even so, the important stunting (WHZ scores lower than − 2) observed in both the LBW and NBW group at birth and up to 12 months of age suggests that nutrition and feeding practices are a challenge in Haiti. This was described previously in Haiti and was thought to be due to a breakdown in the parent-child relationship [30]. Stunting has been repeatedly shown to be a risk factor for delayed neurodevelopment. A meta-analysis in infants across 15 low and middle income (LMIC) countries showed the negative association between stunting and development [31]. A recent study in Rwanda also showed that LBW and NBW babies that were stunted were significantly more likely to experience developmental delay compared to non-stunted NBW babies [32].

The Bayley III scale is a protocolized manner of measuring the neurodevelopment of our study cohort. Across all categories (motor, cognitive and communication), NBW babies were estimated to have higher raw scores compared to LBW babies for the duration of the study period. An unexpected finding was that NBW and LBW babies were estimated to perform similarly at all age groups for fine motor skills, but larger differences existed for the estimated gross motor skills in the same age groups. We are unable to explain this observation but hypothesize that it might have to do with the sequence in which Haitian children acquire gross and motor skills (which might differ in other countries). Larger differences between NBW and LBW were estimated for cognitive and communication skills, with the largest proportional differences estimates for expressive communication skills (and NBW babies performing better in these categories). This suggests that the cognitive development and communication of LBW babies in Haitian society are delayed. This is not unexpected and has been well described in other studies [33].

One unexpected finding from this study was that male babies had consistently higher estimates for weight, length, cognitive skills and communication skills in both the NBW and LBW groups. In contrast, most studies report that female infants consistently outperform their male counterparts in both development and mortality. Two recent studies (one in Slovenia and Croatia and one in South Africa), showed that females consistently outperformed male infants in Bayley III cognitive and communication skills [34, 35]. Also, globally it has been shown that female preterm babies have better overall mortality outcomes than male babies [36]. The hypothesis for this being that immunological responses (both innate and adaptive) are different between male and female babies and that these determine how they respond to disease and their overall clinical outcomes [37]. We were unable to find much evidence in line with this specific finding. Studies from South Asia in the 1970s and 1980s showed that female infant mortality exceeded that of males in the age group in the second half of their first year of life [38, 39]. These findings were explained by a behavioral preference for nutrition and healthcare of male babies, possibly influenced by having to make selective decisions of the allocation of scarce resources (39,40). In Haiti, our study team reported anecdotally, that parental interactions with male babies are preferred over female babies, both in terms of attention by the mother and surrounding persons. This remains speculative, but this would also suggest that in the Haiti environment, in terms of nutrition and cognitive and communication stimulus, male babies would advance more compared to females.

This study faced numerous limitations. The unexpected high mortality in the LBW baby group due to the hospital acquired outbreak in the neonatal care unit, probably meant that a large proportion of the most vulnerable LBW babies did not survive for the remainder of the study period. The results of this study therefore cannot be extrapolated to all LBW babies, as the study group likely represents the ‘stronger’ babies that were enrolled in the cohort. Also, partially due to this high mortality, we did not achieve the original calculated study sample size. Considering the narrow confidence intervals obtained from our estimates, we do not think this impacted on the power of our study. We were unable to reliably adjust for prematurity in the models for physiological growth and neurodevelopment as gestational age measurements in Haiti were done through a variety of methods and were not standardized. This limitation is well acknowledged in similar studies in low resource settings as distinguishing between LBW, SGA and prematurity (or a combination of these) where unreliable gestational age measurements is difficult (41). There are no validated Bayley III scales for Haiti, thus we were unable to compare the scores of our study cohort to a standardized population and the comparison could only be done between the NBW and LBW groups. The sample size requirements to validate the Bayley III scales for the Haitian population were too large to be feasible to explore in the current study. We also we unable to calculate reliably the intra and inter-reliability between study teams for their neurodevelopmental assessments. Even though we minimized differences by conducting quality control checks and discussing discrepancies, we could not account for these in our final analysis. Finally, for the Bayley III scales regression models, we only had three or four follow up visit data from the study cohort. This limited the ability to introduce more knots into the regression models which might have led to the current estimates being quite crude estimates of neurodevelopment in Haitian infants. Even so, we are confident that the differences estimated between the LBW and NBW are reliable.

## Conclusion

LBW babies that survive any inpatient neonatal care in Haiti and live up to 24 months of age, will almost catch up to their NBW baby peers in terms of weight, length and fine motor skills. There does appear to be a delay in the LBW group in terms of gross motor, cognitive and communication skills development. Even though such delays are known to cause cognitive and behavioral problems in the future in other settings, the precise impact of these delays in the Haitian context would merit further research. Also, validating neurodevelopmental tools such as the Bayley scales for the Haitian pediatric population would be of added value, to better understand the clinical significance of delays observed. We will also further explore the current study data to identify specific maternal or hospital related interventions that impact positively on growth and neurodevelopment of LBW babies in order to better guide clinical management of LBW infants in low resource contexts.

## Supplementary Information

**Additional file 1: Figure S1**: Weight measurements in grams by NBW and LBW babies over 24 month follow up period. **Figure S2**: Weight gain (g per day per kg from previous visit) in the NBW and LBW babies over 24 month follow up period. **Figure S3**: Height measurements in cm for NBW and LBW babies over 24 month follow up period. **Table S 1** Weight change per day (in g) from the previous visit by group (NBW vs LBW). **Table S2** Estimated weight from linear mixed models and 95% confidence intervals (in grams) at birth, 6, 12, 18 and 24 months for NBW and LBW infants adjusted by age and sex. **Table S3** Estimated length from linear mixed models and 95% confidence intervals (in grams) at birth, 6, 12, 18 and 24 months for NBW and LBW infants adjusted by sex. **Table S4** Estimated Bayley Scales III raw scores from linear mixed models and 95% confidence intervals for gross motor skills, fine motor skills, cognitive skills, receptive communication skills and expressive communication skills in female infants. **Table S5** Estimated Bayley Scales III raw scores from linear mixed models and 95% confidence intervals for gross motor skills, fine motor skills, cognitive skills, receptive communication skills and expressive communication skills in male infants. **Table S6** Prediction models for outcome weight and length with predictor of age. **Table S7**: Prediction models for outcome weight and length with predictor of age and sex. **Table S8**: Prediction models for outcome weight and length with predictor of age, sex and prematurity. **Table S9**: Prediction models for cognitive, receptive communication and expressive communication skills with predictor of age. **Table S10**: Prediction models for cognitive, receptive communication and expressive communication skills with predictor of age and sex. **Table S11**: Prediction models for gross and fine motor skills with predictor of age. **Table S12**: Prediction models for gross and fine motor skills with predictor of age and sex. **Table S13**: Prediction models for gross and fine motor skills with predictor of age, sex and prematurity. **Table S14**: Reference list of the R packages used in the data analysis.

## Data Availability

MSF has a managed access system for data sharing. Data are available on request in accordance with MSF’s data sharing policy. Requests for access to data should be made to data.sharing@msf.org. For more information please see: 1. MSF’s Data Sharing Policy: http://fieldresearch.msf.org/msf/handle/10144/306501 2. MSF’s Data Sharing Policy PLOS Medicine article: http://journals.plos.org/plosmedicine/article?id=10.1371/journal.pmed.1001562

## Abbreviations

BCG: Bacillus Calmette Guérin (vaccine)
CPAP: Continuous positive airway pressure
CRUO: Centre de Référence des Urgences Obstétricales
ELBW: Extreme low birthweight
KMC: Kangaroo Mother Care
LBW: Low birthweight
LMIC: Low and middle income countries
NBW: Normal birthweight
NGT: Nasogastric tube
NLS: Neonatal life support
SD: Standard deviation
SGA: Small for gestational age
VLBW: Very low birthweight
WHZ: Weight for Height z-scores
95%CI: 95% Confidence Intervals
