# Supplementary material for: Growth and neurodevelopment in low birth weight versus normal birth weight infants from birth to 24 months, born in an obstetric emergency hospital in Haiti, a prospective cohort study
Source: BMC Pediatr. 2021 Mar 24;21:143. doi: 10.1186/s12887-021-02605-3 (PMC7988959; doi:10.1186/s12887-021-02605-3)
Supplement: Supplementary file 1 — Additional file 1: Figure S1: Weight measurements in grams by NBW and LBW babies over 24 month follow up period. Figure S2: Weight gain (g per day per kg from previous visit) in the NBW and LBW babies over 24 month follow up period. Figure S3: Height measurements in cm for NBW and LBW babies over 24 month follow up period. Table S 1 Weight change per day (in g) from the previous visit by group (NBW vs LBW). Table S2 Estimated weight from linear mixed models and 95% confidence intervals (in grams) at birth, 6, 12, 18 and 24 months for NBW and LBW infants adjusted by age and sex. Table S3 Estimated length from linear mixed models and 95% confidence intervals (in grams) at birth, 6, 12, 18 and 24 months for NBW and LBW infants adjusted by sex. Table S4 Estimated Bayley Scales III raw scores from linear mixed models and 95% confidence intervals for gross motor skills, fine motor skills, cognitive skills, receptive communication skills and expressive communication skills in female infants. Table S5 Estimated Bayley Scales III raw scores from linear mixed models and 95% confidence intervals for gross motor skills, fine motor skills, cognitive skills, receptive communication skills and expressive communication skills in male infants. Table S6 Prediction models for outcome weight and length with predictor of age. Table S7: Prediction models for outcome weight and length with predictor of age and sex. Table S8: Prediction models for outcome weight and length with predictor of age, sex and prematurity. Table S9: Prediction models for cognitive, receptive communication and expressive communication skills with predictor of age. Table S10: Prediction models for cognitive, receptive communication and expressive communication skills with predictor of age and sex. Table S11: Prediction models for gross and fine motor skills with predictor of age. Table S12: Prediction models for gross and fine motor skills with predictor of age and sex. Table S13: Prediction models for gross and fi [file 12887_2021_2605_MOESM1_ESM.docx]

# Supplementary Information

Figure S1: Weight measurements in grams by NBW and LBW babies over 24 month follow up period.


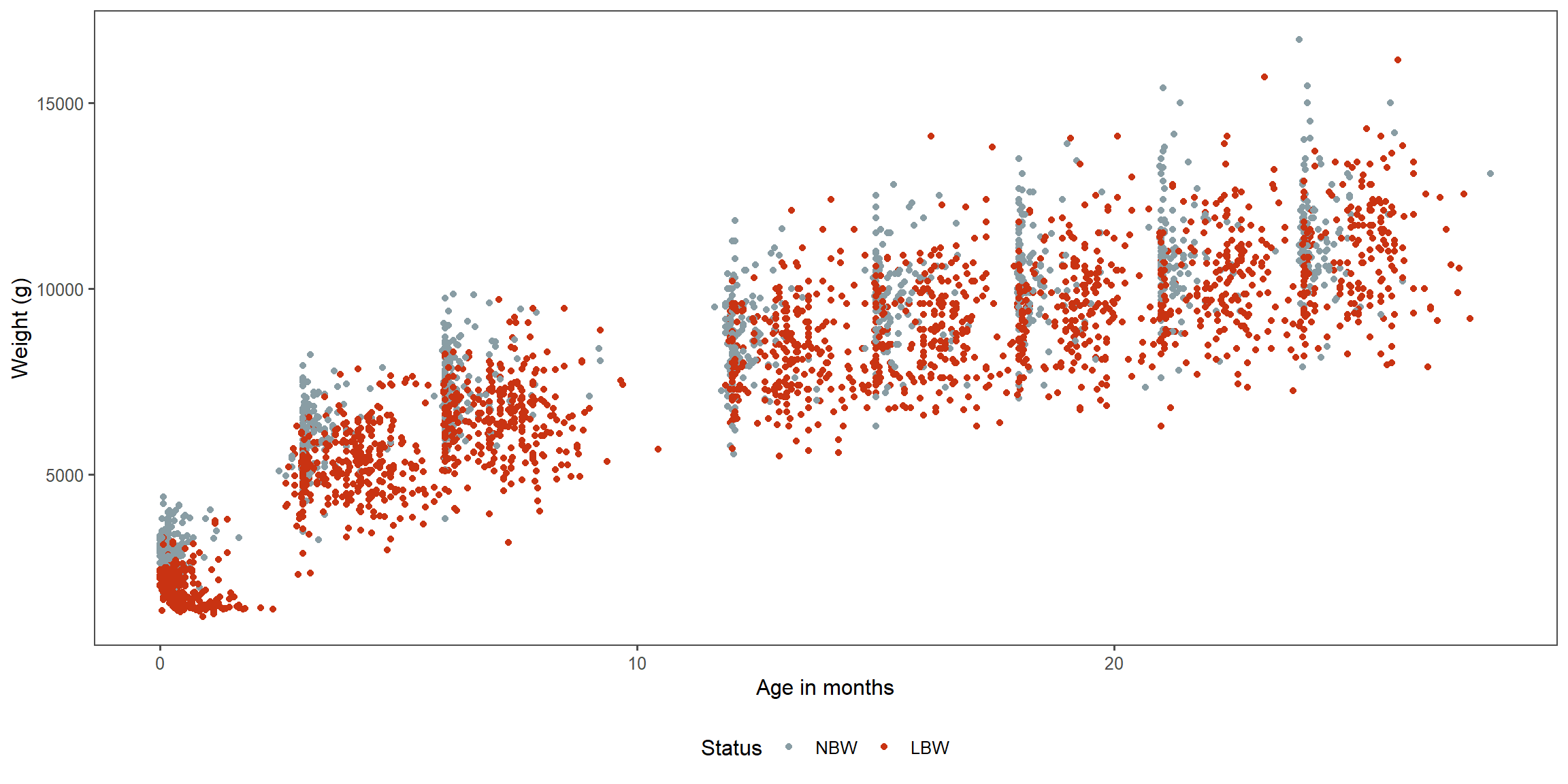


Figure S2: Weight gain (g per day per kg from previous visit) in the NBW and LBW babies over 24 month follow up period.


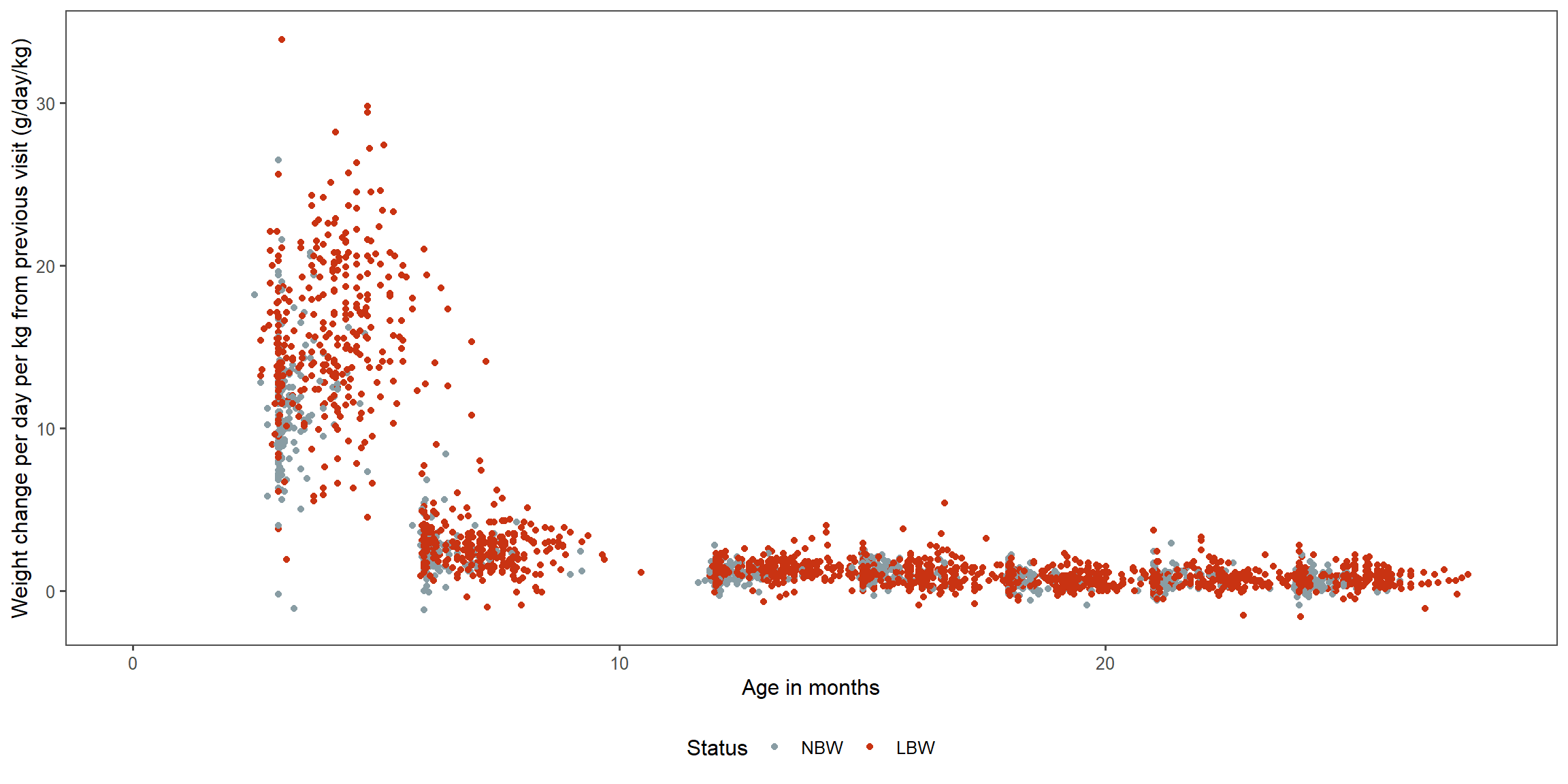


Table S 1 Weight change per day (in g) from the previous visit by group (NBW vs LBW).

|  | **Weight change per day from the previous visit (g)** | | | |
| --- | --- | --- | --- | --- |
|  | **NBW** | | **LBW** | |
| **visit** | **n** | **Mean (sd)** | **n** | **Mean (sd)** |
| **3** | **197** | 33.3 (8.2) | **376** | 30.2 (7.5) |
| **6** | **179** | 14.7 (6.3) | **332** | 13.7 (6.1) |
| **12** | **164** | 7 (3.4) | **312** | 8.6 (3.6) |
| **15** | **151** | 10.2 (4.3) | **273** | 8.4 (5.8) |
| **18** | **143** | 6.2 (4.9) | **251** | 6.2 (4.4) |
| **21** | **135** | 6.8 (5.2) | **236** | 7.2 (5.4) |
| **24** | **127** | 5.8 (5.5) | **218** | 7.8 (5.5) |
|  |  |  |  |  |
| **visit** | **n** | **Median [range]** | **n** | **Median [range]** |
| **3** | **197** | 32.6 [-3.7-56.2] | **376** | 30.5 [3.9-49.9] |
| **6** | **179** | 14.1 [-6.8-33.1] | **332** | 13.5 [-6.7-36.8] |
| **12** | **164** | 6.5 [-1.8-14.9] | **312** | 8.5 [-4.6-24.7] |
| **15** | **151** | 10.3 [-2.7-20] | **273** | 8 [-7.4-40.7] |
| **18** | **143** | 5.9 [-7.7-21.6] | **251** | 6.1 [-5.4-20.5] |
| **21** | **135** | 6.1 [-7.1-23.5] | **236** | 6.6 [-17.8-31.5] |
| **24** | **127** | 5.3 [-11.1-24] | **218** | 7.5 [-14.6-29.7] |

*Table S2 Estimated weight from linear mixed models and 95% confidence intervals (in grams) at birth, 6, 12, 18 and 24 months for NBW and LBW infants adjusted by age and sex.*

|  |  | **NBW** | | **LBW** | |
| --- | --- | --- | --- | --- | --- |
|  | **Age (months)** | **Estimated weight (g)** | **95%CI** | **Estimated weight (g)** | **95%CI** |
| **Females** | **0** | 2679.3 | 2607.4- 2751.1 | 1507.0 | 1400.5-1613.6 |
|  | **6** | 6946.4 | 6803.5- 7089.2 | 5893.2 | 5744.9-6041.5 |
|  | **12** | 8772.2 | 8623.9- 8920.5 | 7929.5 |  |
|  | **18** | 10016.7 |  | 9183.8 |  |
|  | **24** | 11261.1 |  | 10438.0 |  |
|  | | | | | |
| **Males** | **0** | 2851.3 | 2754.9-2947.7 | 1679.07 | 1532.4-1825.8 |
|  | **6** | 7118.4 | 6922.4-7314.5 | 6065.24 | 5861.1-6269.4 |
|  | **12** | 8944.2 | 8712.3-9176.2 | 8101.58 |  |
|  | **18** | 10188.7 |  | 9355.80 |  |
|  | **24** | 11433.1 |  | 10610.02 |  |

**NBW = normal birth weight, LBW = low birth weight**

Figure S3: Height measurements in cm for NBW and LBW babies over 24 month follow up period.


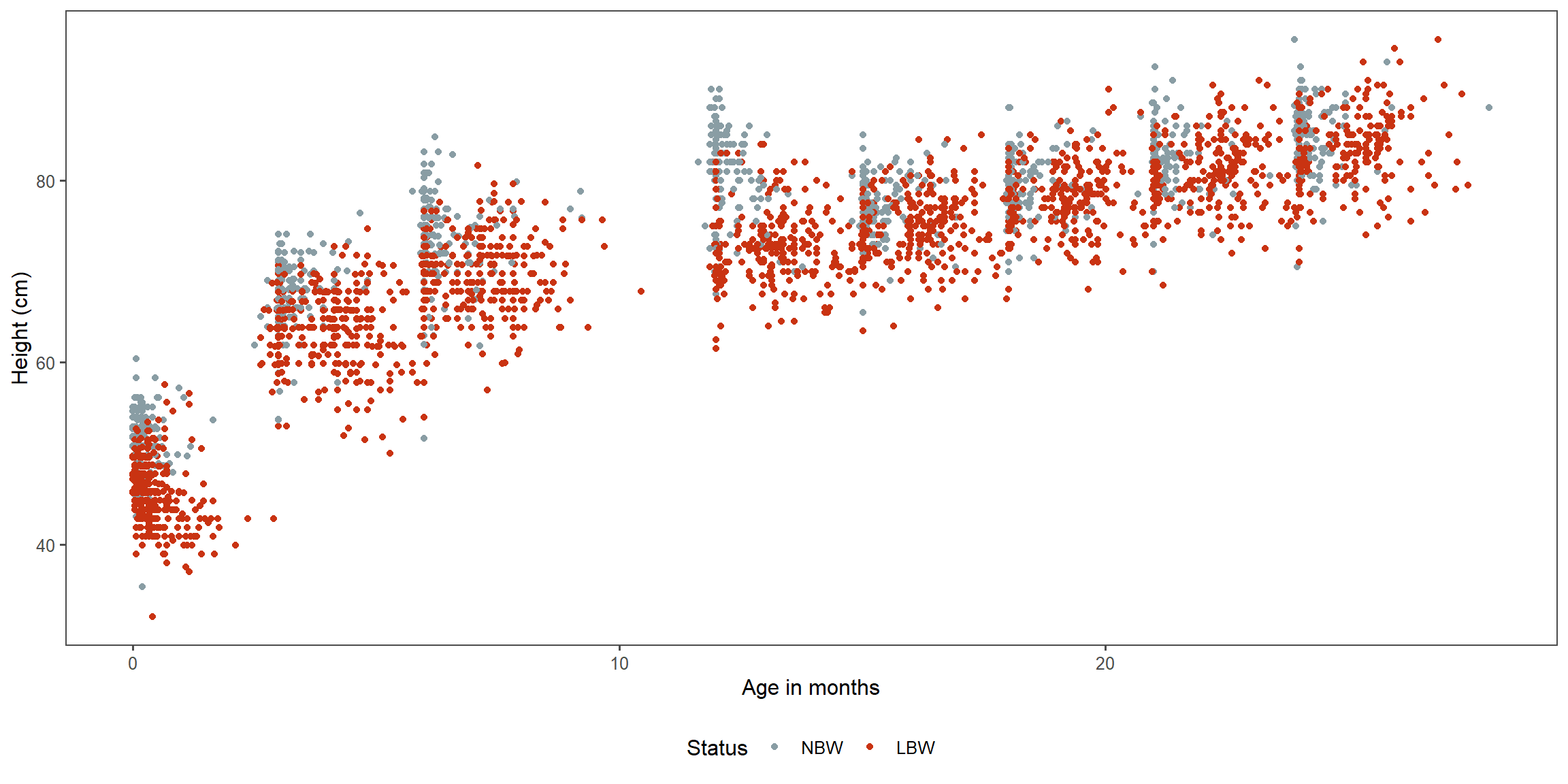


*Table S3 Estimated length from linear mixed models and 95% confidence intervals (in grams) at birth, 6, 12, 18 and 24 months for NBW and LBW infants adjusted by sex.*

|  |  | **NBW** | | **LBW** | |
| --- | --- | --- | --- | --- | --- |
|  | **Age (months)** | **Estimated length (cm)** | **95%CI** | **Estimated length (cm)** | **95%CI** |
| **Females** | **0** | 49.5 | 49.0- 50.0 | 43.06 | 42.6- 43.5 |
|  | **6** | 70.6 | 70.1- 71.1 | 65.81 | 65.4- 66.3 |
|  | **12** | 78.0 | 77.5- 78.5 | 72.03 |  |
|  | **18** | 80.2 |  | 76.70 |  |
|  | **24** | 82.5 |  | 81.36 |  |
|  | | | | | |
| **Males** | **0** | 50.6 | 49.9- 51.2 | 44.17 | 43.6- 44.8 |
|  | **6** | 71.7 | 71.0- 72.4 | 66.92 | 66.3- 67.6 |
|  | **12** | 79.1 | 78.4- 79.8 | 73.14 |  |
|  | **18** | 81.3 |  | 77.81 |  |
|  | **24** | 83.6 |  | 82.47 |  |

**NBW = normal birth weight, LBW = low birth weight**

Table S4 Estimated Bayley Scales III raw scores from linear mixed models and 95% confidence intervals for gross motor skills, fine motor skills, cognitive skills, receptive communication skills and expressive communication skills in female infants.

| **Bayley scale** |  | **NBW** |  | **LBW** |  |
| --- | --- | --- | --- | --- | --- |
|  | **Age (months)** | **Estimated score** | **95%CI** | **Estimated score** | **95%CI** |
| **Gross Motor skills** | **6** | 25.4 | 24.5-26.3 | 21.0 | 20.1-21.9 |
|  | **12** | 40.4 | 39.6-41.1 | 37.2 | 36.3-38.0 |
|  | **18** | 47.4 |  | 44.7 |  |
|  | **24** | 54.4 |  | 52.2 |  |
|  | | | | | |
| **Fine motor skills** | **6** | 19.3 | 18.8-19.9 | 17.4 | 16.9-17.9 |
|  | **12** | 28.9 | 24.5-29.3 | 28.8 | 28.3-29.2 |
|  | **18** | 32.8 |  | 32.3 |  |
|  | **24** | 36.8 |  | 35.8 |  |
|  | | | | | |
| **Cognitive skills** | **12** | 38.4 | 37.2-39.5 | 37.6 | 36.4-38.8 |
|  | **18** | 49.6 | 48.5-50.7 | 47.6 |  |
|  | **24** | 55.4 |  | 55.4 |  |
|  | | | | | |
| **Receptive communication skills** | **12** | 12.8 | 12.3-13.4 | 11.7 | 11.1-12.2 |
|  | **18** | 18.2 | 17.7-18.6 | 17.4 |  |
|  | **24** | 23.8 |  | 22.4 |  |
|  | | | | | |
| **Expressive communication skills** | **12** | 12.3 | 11.5-13.1 | 11.9 | 11.1-12.7 |
|  | **18** | 18.2 | 17.5-18.9 | 16.2 |  |
|  | **24** | 24.9 |  | 22.6 |  |

Table S5 Estimated Bayley Scales III raw scores from linear mixed models and 95% confidence intervals for gross motor skills, fine motor skills, cognitive skills, receptive communication skills and expressive communication skills in male infants.

| **Bayley scale** |  | **NBW** |  | **LBW** |  |
| --- | --- | --- | --- | --- | --- |
|  | **Age (months)** | **Estimated score** | **95%CI** | **Estimated score** | **95%CI** |
| **Gross Motor skills** | **6** | 25.9 | 24.7-27.1 | 21.4 | 20.3-22.6 |
|  | **12** | 40.8 | 39.7-41.9 | 37.6 | 36.4-38.8 |
|  | **18** | 47.8 |  | 45.1 |  |
|  | **24** | 54.9 |  | 52.6 |  |
|  | | | | | |
| **Fine motor skills** | **6** | 19.4 | 18.7-20.1 | 17.5 | 16.8-18.1 |
|  | **12** | 29.0 | 28.4-29.5 | 28.8 | 28.1-29.5 |
|  | **18** | 32.9 |  | 32.4 |  |
|  | **24** | 36.9 |  | 35.9 |  |
|  | | | | | |
| **Cognitive skills** | **12** | 39.7 | 38.2-41.2 | 39.0 | 37.4-40.5 |
|  | **18** | 50.9 | 49.4-52.4 | 48.6 |  |
|  | **24** | 56.8 |  | 54.7 |  |
|  | | | | | |
| **Receptive communication skills** | **12** | 12.9 | 12.2-13.6 | 11.7 | 11.0-12.4 |
|  | **18** | 18.2 | 17.6-18.9 | 17.5 |  |
|  | **24** | 23.8 |  | 22.4 |  |
|  | | | | | |
| **Expressive communication skills** | **12** | 12.6 | 11.6-13.6 | 12.2 | 11.2-13.3 |
|  | **18** | 18.6 | 17.6-19.6 | 16.5 |  |
|  | **24** | 25.2 |  | 23.0 |  |

Table S6 Prediction models for outcome weight and length with predictor of age

| TermMeaning | Value | Estimate.weight | Pval.weight | Estimate.length | Pval.length |
| --- | --- | --- | --- | --- | --- |
| Intercept | NA | 2777.4 | 0.000 | 50.1 | 0 |
| Change LBW vs NBW | 1 if LBW | -1199.3 | 0.000 | -6.6 | 0 |
| Monthly change m0-3 | x (x<3); 3 (x>3) | 1117.8 | 0.000 | 5.8 | 0 |
| Monthly change after m3 until m12 | x-3 (x>3 and x=<12) or 12-3 (x>12) | 304.6 | 0.000 | 1.2 | 0 |
| Monthly change after m12 | x-12 (x>12) | 207.3 | 0.000 | 0.4 | 0 |
| if LBW: Monthly change m0-3 | 1 if LBW AND x (x<3); 3 (x>3) | 4.6 | 0.874 | 0.7 | 0 |
| if LBW: Monthly change after m3 until m12 | 1 if LBW AND x-3 (x>3 and x=<12) or 12-3 (x>12) | 34.7 | 0.002 | -0.2 | 0 |
| if LBW: Monthly change after m12 | 1 if LBW AND x-12 (x>12) | 1.7 | 0.815 | 0.4 | 0 |

Table S7: Prediction models for outcome weight and length with predictor of age and sex

| TermMeaning | Value | Estimate.weight | Pval.weight | Estimate.length | Pval.length |
| --- | --- | --- | --- | --- | --- |
| Intercept | NA | 2679.2 | 0.000 | 49.5 | 0 |
| Change LBW vs NBW | 1 if LBW | -1172.2 | 0.000 | -6.4 | 0 |
| Monthly change m0-3 | x (x<3); 3 (x>3) | 1118.1 | 0.000 | 5.8 | 0 |
| Monthly change after m3 until m12 | x-3 (x>3 and x=<12) or 12-3 (x>12) | 304.3 | 0.000 | 1.2 | 0 |
| Monthly change after m12 | x-12 (x>12) | 207.4 | 0.000 | 0.4 | 0 |
| Male vs Female | 1 if male | 172.0 | 0.000 | 1.1 | 0 |
| if LBW: Monthly change m0-3 | 1 if LBW AND x (x<3); 3 (x>3) | 4.6 | 0.873 | 0.7 | 0 |
| if LBW: Monthly change after m3 until m12 | 1 if LBW AND x-3 (x>3 and x=<12) or 12-3 (x>12) | 35.1 | 0.002 | -0.2 | 0 |
| if LBW: Monthly change after m12 | 1 if LBW AND x-12 (x>12) | 1.6 | 0.819 | 0.4 | 0 |

Table S8: Prediction models for outcome weight and length with predictor of age, sex and prematurity

| TermMeaning | Value | Estimate.weight | Pval.weight | Estimate.length | Pval.length |
| --- | --- | --- | --- | --- | --- |
| Intercept | NA | 2763.9 | 0.000 | 49.8 | 0 |
| Change LBW vs NBW | 1 if LBW | -855.5 | 0.000 | -5.1 | 0 |
| Monthly change m0-3 | x (x<3); 3 (x>3) | 1116.8 | 0.000 | 5.8 | 0 |
| Monthly change after m3 until m12 | x-3 (x>3 and x=<12) or 12-3 (x>12) | 304.0 | 0.000 | 1.2 | 0 |
| Monthly change after m12 | x-12 (x>12) | 207.5 | 0.000 | 0.4 | 0 |
| Male vs Female | 1 if male | 118.1 | 0.004 | 1.0 | 0 |
| Change preterm vs fullterm | 1 if preterm | -620.7 | 0.000 | -2.5 | 0 |
| if LBW: Monthly change m0-3 | 1 if LBW AND x (x<3); 3 (x>3) | 5.6 | 0.846 | 0.7 | 0 |
| if LBW: Monthly change after m3 until m12 | 1 if LBW AND x-3 (x>3 and x=<12) or 12-3 (x>12) | 35.4 | 0.002 | -0.2 | 0 |
| if LBW: Monthly change after m12 | 1 if LBW AND x-12 (x>12) | 1.5 | 0.831 | 0.4 | 0 |

Table S9: Prediction models for cognitive, receptive communication and expressive communication skills with predictor of age

| TermMeaning | Value | Estimate.CS | Pval.CS | Estimate.RC | Pval.RS | Estimate.EC | Pval.EC |
| --- | --- | --- | --- | --- | --- | --- | --- |
| Intercept | NA | 16.7 | 0.000 | 2.2 | 0.016 | 0.5 | 0.675 |
| Change LBW vs NBW | 1 if LBW | 2.1 | 0.431 | -2.1 | 0.100 | 2.9 | 0.108 |
| Monthly change m12-18 | x (x<18); 18 (x>18) | 1.9 | 0.000 | 0.9 | 0.000 | 1.0 | 0.000 |
| Monthly change after m18 | x-18 (x>18) | 1.0 | 0.000 | 0.9 | 0.000 | 1.1 | 0.000 |
| if LBW: Monthly change m12-18 | 1 if LBW AND x (x<18); 18 (x>18) | -0.3 | 0.132 | 0.1 | 0.370 | -0.3 | 0.017 |
| if LBW: Monthly change after m18 | 1 if LBW AND x-18 (x>18) | 0.0 | 0.771 | -0.1 | 0.118 | 0.0 | 0.703 |

Table S10: Prediction models for cognitive, receptive communication and expressive communication skills with predictor of age and sex

| TermMeaning | Value | Estimate.CS | Pval.CS | Estimate.RC | Pval.RS | Estimate.EC | Pval.EC |
| --- | --- | --- | --- | --- | --- | --- | --- |
| Intercept | NA | 15.9 | 0.000 | 2.1 | 0.020 | 0.3 | 0.792 |
| Change LBW vs NBW | 1 if LBW | 2.4 | 0.369 | -2.0 | 0.103 | 2.9 | 0.100 |
| Monthly change m12-18 | x (x<18); 18 (x>18) | 1.9 | 0.000 | 0.9 | 0.000 | 1.0 | 0.000 |
| Monthly change after m18 | x-18 (x>18) | 1.0 | 0.000 | 0.9 | 0.000 | 1.1 | 0.000 |
| Male vs Female | 1 if male | 1.3 | 0.037 | 0.1 | 0.806 | 0.3 | 0.423 |
| if LBW: Monthly change m12-18 | 1 if LBW AND x (x<18); 18 (x>18) | -0.3 | 0.131 | 0.1 | 0.369 | -0.3 | 0.017 |
| if LBW: Monthly change after m18 | 1 if LBW AND x-18 (x>18) | 0.0 | 0.770 | -0.1 | 0.118 | 0.0 | 0.703 |

Table S10: Prediction models for cognitive, receptive communication and expressive communication skills with predictor of age, sex and prematurity

| TermMeaning | Value | Estimate.CS | Pval.CS | Estimate.RC | Pval.RS | Estimate.EC | Pval.EC |
| --- | --- | --- | --- | --- | --- | --- | --- |
| Intercept | NA | 16.0 | 0.000 | 2.2 | 0.015 | 0.5 | 0.710 |
| Change LBW vs NBW | 1 if LBW | 3.0 | 0.268 | -1.5 | 0.239 | 3.7 | 0.040 |
| Monthly change m12-18 | x (x<18); 18 (x>18) | 1.9 | 0.000 | 0.9 | 0.000 | 1.0 | 0.000 |
| Monthly change after m18 | x-18 (x>18) | 1.0 | 0.000 | 0.9 | 0.000 | 1.1 | 0.000 |
| Change male vs female | 1 if male | 1.3 | 0.043 | 0.0 | 0.911 | 0.3 | 0.495 |
| Change preterm vs fullterm | 1 if preterm | -1.3 | 0.096 | -1.2 | 0.000 | -1.6 | 0.001 |
| if LBW: Monthly change m12-18 | 1 if LBW AND x (x<18); 18 (x>18) | -0.3 | 0.143 | 0.1 | 0.323 | -0.3 | 0.021 |
| if LBW: Monthly change after m18 | 1 if LBW AND x-18 (x>18) | 0.0 | 0.746 | -0.1 | 0.137 | 0.0 | 0.750 |

Table S11: Prediction models for gross and fine motor skills with predictor of age

| TermMeaning | Value | Estimate.fm | Pval.fm | Estimate.gm | Pval.gm |
| --- | --- | --- | --- | --- | --- |
| Intercept | NA | 9.8 | 0.000 | 10.8 | 0.000 |
| Change LBW vs NBW | 1 if LBW | -3.7 | 0.000 | -5.7 | 0.000 |
| Monthly change m6-12 | x (x<12); 12 (x>12) | 1.6 | 0.000 | 2.5 | 0.000 |
| Monthly change after m12 | x-12 (x>12) | 0.7 | 0.000 | 1.2 | 0.000 |
| if LBW: Monthly change m6-12 | 1 if LBW AND x (x<12); 12 (x>12) | 0.3 | 0.000 | 0.2 | 0.041 |
| if LBW: Monthly change after m12 | 1 if LBW AND x-12 (x>12) | -0.1 | 0.049 | 0.1 | 0.116 |

Table S12: Prediction models for gross and fine motor skills with predictor of age and sex

| TermMeaning | Value | Estimate.fm | Pval.fm | Estimate.gm | Pval.gm |
| --- | --- | --- | --- | --- | --- |
| Intercept | NA | 9.8 | 0.000 | 10.5 | 0.000 |
| Change LBW vs NBW | 1 if LBW | -3.7 | 0.000 | -5.7 | 0.000 |
| Monthly change m6-12 | x (x<12); 12 (x>12) | 1.6 | 0.000 | 2.5 | 0.000 |
| Monthly change after m12 | x-12 (x>12) | 0.7 | 0.000 | 1.2 | 0.000 |
| Male vs Female | 1 if male | 0.1 | 0.785 | 0.4 | 0.397 |
| if LBW: Monthly change m6-12 | 1 if LBW AND x (x<12); 12 (x>12) | 0.3 | 0.000 | 0.2 | 0.039 |
| if LBW: Monthly change after m12 | 1 if LBW AND x-12 (x>12) | -0.1 | 0.049 | 0.1 | 0.116 |

Table S13: Prediction models for gross and fine motor skills with predictor of age, sex and prematurity

| TermMeaning | Value | Estimate.fm | Pval.fm | Estimate.gm | Pval.gm |
| --- | --- | --- | --- | --- | --- |
| Intercept | NA | 13.1 | 0.000 | 14.1 | 0.000 |
| Change LBW vs NBW | 1 if LBW | -1.9 | 0.001 | -3.7 | 0.000 |
| Monthly change m12-18 | x (x<18); 18 (x>18) | 1.2 | 0.000 | 2.0 | 0.000 |
| Monthly change after m18 | x-18 (x>18) | 0.4 | 0.000 | 0.5 | 0.000 |
| Change male vs female | 1 if male | 0.0 | 0.953 | 0.3 | 0.620 |
| Change preterm vs fullterm | 1 if preterm | -0.3 | 0.340 | -1.8 | 0.005 |
| if LBW: Monthly change m12-18 | 1 if LBW AND x (x<18); 18 (x>18) | 0.1 | 0.010 | 0.1 | 0.067 |
| if LBW: Monthly change after m18 | 1 if LBW AND x-18 (x>18) | -0.1 | 0.140 | 0.2 | 0.018 |

Table S14: Reference list of the R packages used in the data analysis

| 1. Douglas Bates, Martin Maechler, Ben Bolker, Steve Walker (2015).  Fitting Linear Mixed-Effects Models Using lme4. Journal of Statistical Software, 67(1), 1-48. doi:10.18637/jss.v067.i01. 2. Lüdecke D (2021). _sjPlot: Data Visualization for Statistics in Social Science_. R package version 2.8.7, <URL: <https://CRAN.R-project.org/package=sjPlot>>. 3. Lüdecke D (2018). "ggeffects: Tidy Data Frames of Marginal Effects from Regression Models." _Journal of Open Source Software_, *3*(26), 772. doi: 10.21105/joss.00772 (URL: <https://doi.org/10.21105/joss.00772>). 4. Kuznetsova A, Brockhoff PB, Christensen RHB (2017). "lmerTest Package: Tests in Linear Mixed Effects Models." _Journal of Statistical Software_, *82*(13), 1-26. doi: 10.18637/jss.v082.i13 (URL: <https://doi.org/10.18637/jss.v082.i13>). 5. Wickham et al., (2019). Welcome to the tidyverse. Journal of Open Source Software, 4(43), 1686, <https://doi.org/10.21105/joss.01686> 6. Russell V. Lenth (2020). emmeans: Estimated Marginal Means, aka Least-Squares Means. R package version 1.5.3.  <https://CRAN.R-project.org/package=emmeans> 7. Michal Bojanowski (2017). lspline: Linear Splines with Convenient Parametrisations. R package version 1.0-0. <https://CRAN.R-project.org/package=lspline> 8. Jeffrey B. Arnold (2021). ggthemes: Extra Themes, Scales and Geoms for 'ggplot2'. R package version 4.2.4.  <https://CRAN.R-project.org/package=ggthemes> 9. Karthik Ram and Hadley Wickham (2018). wesanderson: A Wes Anderson Palette Generator. R package version 0.3.6. <https://CRAN.R-project.org/package=wesanderson> 10. Yihui Xie (2021). knitr: A General-Purpose Package for Dynamic Report Generation in R. R package version 1.31. 11. Yihui Xie (2015) Dynamic Documents with R and knitr. 2nd edition. Chapman and Hall/CRC. ISBN 978-1498716963 12. Yihui Xie (2014) knitr: A Comprehensive Tool for Reproducible   Research in R. In Victoria Stodden, Friedrich Leisch and Roger D.  Peng, editors, Implementing Reproducible Computational Research.  Chapman and Hall/CRC. ISBN 978-1466561595 |
| --- |
